# The cost-effectiveness of specialist hospital discharge and intermediate care services for patients who are homeless

**DOI:** 10.1186/s12913-025-12704-x

**Published:** 2025-06-03

**Authors:** Michela Tinelli, Raphael Wittenberg, Michelle Cornes, Robert W. Aldridge, Michael Clark, Richard Byng, Graham Foster, James Fuller, Andrew Hayward, Nigel Hewett, Alan Kilmister, Jill Manthorpe, Joanne Neale, Elizabeth Biswell, Martin Whiteford

**Affiliations:** 1 Care Policy and Evaluation Centre, The London School of Economics and Political Science, Houghton Street, London, WC2A 2AE UK; 2 School of Health and Society, University of Salford, Mary Seacole Building, Frederick Road Campus, Broad St, Frederick Road Campus, Salford, M6 6PU UK; 3 NIHR Policy Research Unit in Health and Social Care Workforce, 1St Floor, Virginia Woolf Building, 22 Kingsway, London, WC2R 2LS UK; 4 Department of Epidemiology and Public Health, Institute of Health Informatics, University College London, Institute of Epidemiology and Health Care, 1 - 19 Torrington Place, London, WCLE 7HB UK; 5 Clinical Trials and Health Research, University of Plymouth, ITTC, Drake Circus, Plymouth, Devon PL4 8AA UK; 6 Blizard Institute, Queen Mary University of London, Mile End Road, London, E1 4NS UK; 7 Pathway and the Faculty for Homeless and Inclusion Health, 4th Floor East, 250 Euston Road, London, NW1 2PG UK; 8 National Addiction Centre, Institute of Psychiatry, Psychology & Neuroscience, King’s College London Addictions Sciences Building, 4 Windsor Walk, Denmark Hill, London, SE58BB UK; 9 Emerald Publishing, Limited Howard House Wagon Lane, Bingley, BD16 1WA UK

**Keywords:** Cost-effectiveness, Economic evaluation, Homelessness, Hospital discharge, Intermediate care

## Abstract

**Background:**

Recognising the diverse healthcare needs of the population, there is a growing emphasis on tailoring hospital discharge processes to address the unique challenges faced by individuals who are homeless, aiming to enhance the efficiency and effectiveness of post-hospitalisation care for this vulnerable demographic. This study aimed to evaluate the costs and consequences of specialist hospital discharge and intermediate care (support after discharge) services for people who are homeless in England.

**Methods:**

We estimated the comparative costs and consequences of different types of specialist care provided by 17 homeless hospital discharge and intermediate care services. We compared ‘clinically-led’ (multidisciplinary) services with those that were ‘housing-led’ (uniprofessional). A retrospective observational study was conducted to estimate effectiveness and costs for two'intervention groups'(clinically-led and housing-led) and a previously published RCT for'standard care'. Use of resources data for specialist care was sourced through linkage with Hospital Episode Statistics. The measure of effectiveness was the number of bed days avoided (in terms of hospital stays for all readmissions in the follow-up period) per homeless user. Additional secondary analysis of three services looked at quality-adjusted life years (QALYs) and service delivery costs. The perspective adopted was NHS in England.

**Results:**

Data from the comparative analysis showed that specialist homeless hospital discharge (HHD) care is likely to be cost-effective compared with standard care. Patients accessing specialist care use fewer bed days per year (including both planned and unplanned readmissions). Patients using specialist care have more planned readmissions to hospital and, overall, use more NHS resources than those who use standard care. We interpret this as a positive outcome indicating that specialist care is likely to work more effectively than standard care to improve access to healthcare for this marginalised group. Specialist care remained cost-effective over a range of sensitivity analyses. Secondary analyses of three specific schemes found better QALY outcomes, but results are not generalisable to all 17 schemes.

**Conclusion:**

Specialist HHD services are likely to be cost-effective for the NHS compared with standard care, although further research is needed to access patient level data for both costs and outcomes to conduct a rigorous statistical analysis between groups and address possible underlying biases due to data coming from non-randomised study design.

**Supplementary Information:**

The online version contains supplementary material available at 10.1186/s12913-025-12704-x.

## Background

The Homelessness Monitor reports that ‘core homelessness’ in England – defined as the most severe and immediate forms of homelessness [1] – is estimated to have reached nearly 220,000 in 2019, having increased from about 187,000 in 2012 [2]. During 2020 these numbers dropped to 200,000, mainly because of the Government’s emergency measures in response to the Coronavirus disease 2019 (COVID- 19) pandemic [2]. In 2020 there were an estimated 10,500 people currently sleeping rough on any given night (it dropped by a third from the previous year) [2]. Even before the COVID- 19 pandemic homelessness in all its forms (e.g., from living with friends to staying in temporary accommodation, to sleeping rough) cost the public sector above £1 billion annually in England alone [3].

In 2010, an economic analysis by the Department of Health [DH, now Department of Health and Social Care, DHSC]’s Office of the Chief Analyst showed that people who are homeless accessed hospital Emergency Departments (EDs) five to seven times more often than the general population and that their average length of stay was almost three times the national average [4]. Annual costs of unplanned hospital stay for people with experience of homelessness were eight times higher than those reported by those owning or renting a house/flat [5]. In 2009 a national audit of people with experience of homelessness showed that only 27% of clients had received any help with their housing before being discharged from their most recent admission to the hospital. In response, the DH announced in 2013 that £10 million in funding would be made available to the voluntary and community sector to work in partnership with the NHS and local councils to develop a range of specialist homeless hospital discharge services (HHDs). A key objective of the ‘*Homeless Hospital Discharge Fund’* (HHDF) was to ensure more provision of suitable ‘step-down’ intermediate care [6].

Intermediate, or ‘step-up/down’, care encapsulates a wide range of admission avoidance and out-of-hospital care services delivering targeted, short-term support to individuals to: prevent inappropriate admission to NHS acute inpatient or continuing care, or long-term residential care; facilitate timely discharge from hospital; and, most importantly, maximise people’s ability to live independently within their communities [7].

### Specialist discharge arrangements for people who are homeless

As part of the HHDF initiative, 52 specialist HHDs were established across England. For this economic evaluation, we focus on two principal scheme types. The first is a ‘housing-led’ (uniprofessional) service, in which the HHD service provides a *‘housing link worker’* who works with patients with experience of homelessness in a hospital. Link workers support the patient to find accommodation and assist with other aspects of discharge planning using their knowledge of local homeless services and resources (e.g., in terms of welfare benefits maximisation, social care referrals, and clothing). Once discharged from the hospital, the link worker will usually continue to provide peripatetic/floating ‘step-down’ time-limited intermediate care (usually six weeks).

The second type of service we focus on is ‘clinically-led’ (multi-disciplinary). These are usually co-located in hospitals that see larger numbers of patients with experience of homelessness (over 200 patients with experience of homelessness per year; http://www.pathway.org.uk/). These services are led by General Practitioners (GPs) or nurses with a ‘special interest’ in homeless health care. The teams comprise a range of staff including housing workers, social workers, occupational therapists and ‘peer navigators’ (people with lived experience of homelessness). They are responsible for discharge coordination and contribute to the clinical management of patients while in hospital. They also provide practical support to challenge stigma, for example, by helping patients to access clean clothes and toiletries. Because of this primary focus on improving the quality of in-patient care, clinically-led teams usually close cases at the point of discharge from the hospital and do not provide step-down care. Some clinically-led schemes can directly refer to specialist homeless, residential step-down care facilities. Barriers to accessing mainstream intermediate care (such as 55 + age limits) and a shortage of specialist services leave many clinically-led schemes without access to step-down for their patients.

The purpose of this study was to evaluate the cost and consequences of 17 specialist hospital discharge and intermediate care (support after discharge) services for people who are homeless in England. It compares ‘clinically-led’ (multidisciplinary) services with ‘housing-led’ (uniprofessional) services and schemes providing access to ‘step-down’ intermediate care with schemes that did not. Also, with secondary analysis, we examine three of the services'quality-adjusted life year (QALY) outcomes and service delivery costs.

## Methods

This economic evaluation was undertaken as part of a wider study to evaluate the HHDF [8]. The wider study (2015–2019) employed multiple methods and was underpinned by realist evaluation [9]. Empirical data collection was designed to test the realist hypothesis that, ‘*Clinically-led multidisciplinary schemes encompassing discharge coordination and step-down will be more effective and cost-effective than either standard care or schemes which lack one or more of these key components.’*

We adopted a cost-consequence analysis (CCA) approach as it shows the total cost of implementing an intervention alongside its consequences. It allows the reader to form their own opinion of the intervention’s relevance and importance in their decision-making context.

Our analysis considered the additional NHS resources (in terms of hospital admissions, elective inpatient stays and other readmissions) invested for improvement in outcomes.

### Comparisons and outcomes

The main analysis comprises a comparative focus on 17 HHD schemes. To test the hypothesis above, the economic evaluation adopted a comparative approach:

- (**Comparison A**) What are the costs and consequences of clinically-led (multi-disciplinary teams) (*n* = 12) versus housing-led (uniprofessional schemes) (*n* = 5) *versus* standard care?
- (**Comparison B**) What are the costs and consequences of schemes directly providing or with direct access to step-down intermediate care (*n* = 9) compared to schemes that do not have access to step-down intermediate care (*n* = 8) *versus* standard care?

For each comparison we assessed the costs and effects of two interventions each compared with each other and with standard care. As the primary outcome for the 17 HHD schemes, we considered the number of bed days avoided in the period following the index hospital spell in which the person was discharged to a HHD scheme. The bed days avoided were mainly related to reduced unplanned hospital admissions of people in clinically- or housing-led schemes (Comparison 1) and schemes with or without step-down (Comparison 2) compared to standard care. They were treated as a proxy for improved quality of life. QALY data (a measure of the value of health outcomes) were not made available for all 17 HHD schemes.

The difference in mean 12-month costs and outcomes was estimated and presented as separate items using vertical bar charts.

Table 1 presents a summary of the key parameters considered in the main analysis.

**Table 1:** Summary of the costs consequences model for the main analysis

Parameter	Description
	[Main analysis—looking at the totality of the 17 schemes]
Question to be answered	What are the costs consequences of specialist hospital discharge and intermediate care (support after discharge) services for people who are homeless in England?
Alternatives compared	The analysis includes a comparative focus on 17 HHD schemes focusing on two key comparisons: Comparison A (1: Clinically-led vs control; 2: Housing-led vs control, ‘*standard care’*; 3: Clinically-led vs housing-led); Comparison B (1: No step-down vs control; 2: Step down either community-based or residential vs control; 3: No step down vs step down) *A real control group was not available, so we used proxy data from an earlier RCT * [10].
Type of economic evaluation	Cost-consequence analysis
Country setting	England
Perspective on costs	NHS
Cost data and sources of evidence	Cost data: use of healthcare resources (number of readmissions); Source of evidence: HES data [11] (intervention groups) and Hewett et al. (control group) [10]. *We consider only the economic consequences on NHS [hospital readmissions] as data on service delivery costs were not made available for the 17 HHD schemes*
The base year for calculating costs/prices	All costs are in 2017 prices (the period over which participants were studied and the resources were used)
Currency unit	British pound (£)
Effectiveness outcomes	Cumulative duration of hospital stays (number of bed days after the index admission). This followed published literature [10]. *We consider only cumulative duration of hospital stays as data on QALY, a measure of the value of health outcomes, were not made available for all 17 HHD schemes*
Effectiveness data and sources of evidence	Intervention groups: We used cohorts of patients with experience of homelessness discharged from hospitals in England to estimate the differential effects of different types and configurations of *‘specialist care’* for the NHS (compared to standard care). Estimates for the intervention groups were extracted from HES data [12]. Standard care: We used published data from the Hewett trial [10] (see Table 2)
Time horizon and discounting	12 months. The cost and consequences were considered for a limited period of 12 months, so no discounting needed
Statistical analysis	The cost and effectiveness of the intervention groups was established conducting a comparative analysis by using summary statistics for the control group and individual-level data for the intervention groups. We did not get access to the original cost and outcome dataset from the trial data (with patient-level information) and we could not test for group differences
Sensitivity analysis	We varied individual costs and outcomes by a given amount (up to ± 50%) and examined the impact on model results

The main gaps in data are in italics

Also, a secondary analysis of three schemes with different service configurations was undertaken and it is presented in supplementary material (Appendix 1). It was conducted because more detailed data, on the costs of the schemes and on their outcomes in terms of health-related quality of life, were available for those three schemes but not for the rest of the schemes.

### Effectiveness evidence and source of data

Data for the intervention groups comprised data extracted from Hospital Episode Statistics [12] on their past hospital admissions (main analysis).

For standard care, we used published data from the Hewett trial [10] (see Table 2).

**Table 2:** Number of completed admissions and bed days during one year after index discharge by Homeless Hospital Discharge (HHD) scheme type, configuration and control group

Patient group	Source of data	No. of elective readmissions per patient [Mean, median (SD)]	No. of emergency re-admissions per patient [Mean, median (SD)]	No. of other re-admissions per patient [Mean, median (SD)]	No. of bed days per patient [Mean, median (SD)]	(no. of patients at index discharge)
**Comparison 1**						
Clinically-led	HES DATA	0.92, 1.36 (7.06)	2.62, 3.89 (4.98)	0.10, 0.15 (0.48)	18.88, 28.01 (52.04)	*N* = 2958
Housing-led	HES DATA	0.55, 0.82 (6.11)	2.24, 3.32 (3.71)	0.12, 0.18 (0.48)	19.25, 28.56 (35.98)	*N* = 1040
Patients with experience of homelessness receiving standard care at discharge (control)	Hewett N et al. [10]	0.16, 0.24 (1.25)	1.25, 1.85 (2.61)	0.13, 0.19 (0.56)	20.8, 30.8 (39.99)	*N* = 204
**Comparison 2**						
Schemes offering patient inreach & discharge coordination (with no step-down)	HES DATA	0.98, 1.45 (7.40)	2.55, 3.78 (4.88)	0.10, 0.15 (0.45)	19.23, 28.53 (53.73)	*N* = 2688
Schemes offering patient inreach & discharge coordination (with step-down either community-based or residential)	HES DATA	0.50, 0.74 (5.45)	2.45, 3.63 (5.26)	0.12, 0.18 (0.53)	18.46, 27.39 (34.93)	*N* = 1310
Patients with experience of homelessness receiving standard care at discharge (control)	Hewett N et al. [10]	0.16, 0.24 (1.25)	1.25, 1.85 (2.61)	0.13, 0.19 (0.56)	20.8, 30.86 (39.99)	*N* = 204

For all metrics, the standard deviation is greater than the mean and values are spread out across the distribution. For each group the median is greater than the mean value, indicating that the distribution is skewed to the right. Control group– Hewett et al. [10] did not collect (retrospective) baseline data for the length of stay and use of healthcare resources (emergency, elective and other types of admissions). Intervention groups– similarly to the control group, length of stay and use of resources data were extracted from the HES data linkage for the follow-up period only. This allowed us to replicate the same statistical analyses adopted by Hewett et al. [10]

### Cost data

Cost data were derived from the use of healthcare resources (and related to the costs of hospital inpatient spells only); estimates for the intervention and standard care groups were extracted from HES data [12] and Hewett et al. [10], respectively. Details on the estimates are reported in Table 2.

The quantities were then multiplied by a set of national average unit costs [13], as follows: Hospital Admissions (average £1,783); Elective inpatient stays (£3,903); and other readmissions (£1,074). Unit costs are reported in Appendix 2 (see Supplementary material).

Please note that our analysis includes only the economic consequences on NHS [readmissions] as service delivery costs were not made available for the 17 HHD schemes. The total costs of health services were then summarised at an aggregated level (e.g., according to the type of readmission costs), for the corresponding periods respectively.

The currency unit is British pound (£) and all costs are at 2017 prices.

### Participants

Participants were adults with experience of homelessness over 18 years of age who had experienced one or more hospital admissions and had been discharged to one of the 17 schemes. Patient were grouped according to those who received specialist care (clinically- or housing-led; including either step-down or no step-down schemes; see intervention groups below) and those who received standard care (control group). Intervention group data for 3882 patients from 17 HHD schemes were collected and linked to hospital episode statistics [12]. The control group included data from 204 individuals who participated in the Hewett trial [10]. Study participants’ age, gender distribution and level of comorbidities are comparable between the intervention and control groups [8, 10].

### Intervention (*‘specialist care’*) groups

For intervention groups with'specialist care', we collected information from 17 HHDs. The HHDs were allocated to the typology groups for [clinical v housing led] and [with and without step-down care]. Patient-level data on the use of NHS resources (in terms of elective readmissions, emergency readmissions and other readmissions) and bed days were extracted from HES considering one year after the introduction of specialist care. The two databases were linked following a protocol [12]. Aggregate-level information was extracted from the data linkage cohort to inform our analyses.

### Control (*‘standard care’*) group

As noted, the control group (‘standard care’) aggregate level data on the use of NHS resources were sourced from Hewett et al. [10]. This RCT looked at the effectiveness of two clinically-led services. In the control arm, patients received a leaflet about local homeless services rather than support from the clinically-led team. In our study protocol, we planned to use data from patients with experience of homelessness admitted to hospitals without an HHD. However, it emerged during the study that the homeless service that was used to identify these patients was working with a patient cohort whose health needs were generally lower than those seen by the HHDs. For this reason, the proxy RCT data was thought to offer a better standard care comparison.

### Time horizon

For intervention groups receiving 'specialist care', demographic data were collected for hospital inpatients (HHD service users) from 1st November 2013 to 30 th November 2016. Patient-level data on the utilization of NHS resources (including elective readmissions, emergency readmissions, and other readmissions) and bed days were subsequently extracted from HES, covering one year after the introduction of specialist care. For control group, costs and outcomes data refer to Hewett trial [10].

The cost and consequences were considered for a limited period of 12 months and no discounting was needed.

### Statistical analysis

Mean and standard deviation (SD) are used to describe yearly estimates of costs and outcomes across the three groups. In Comparison A, we present three sets of data differences: 1) Clinically-led vs control, 2) Housing-led vs control, and 3) Clinically-led vs housing-led. As for Comparison B, we provide data differences for 1) No step down vs control, 2) Step down (either community-based or residential) vs control, and 3) No step down vs step down. We did not get access to the original cost and outcome dataset from the trial data (with patient-level information) and we could not test for group differences between interventions and control. The independent p-value test was used to examine differences between intervention groups. Details on the difference in use of resources and statistically significant results for the trial are presented by Hewett trial [10]. More about HES data used in this modelling is presented elsewhere [14].

### Sensitivity analysis

We looked at individual categories of costs and outcomes, varying their relative estimates in the model by a given amount (up to ± 50%) and examining the impact on model results. Analyses were computed in Excel.

## Results

### The effect of HHD schemes on health care resources and costs (considering data from the 17 HHD schemes)

#### Bed days avoided per patient

When looking at mean values, patients using an HHD scheme used slightly fewer bed days (during one year after index discharge) than patients discharged from the hospital without the support of an HHD scheme. But please note that the standard deviation is much greater than the mean and values are spread out across the distribution. Details on the mean, median and standard deviation values for each group are provided in Table 2.

**Fig. 1:**
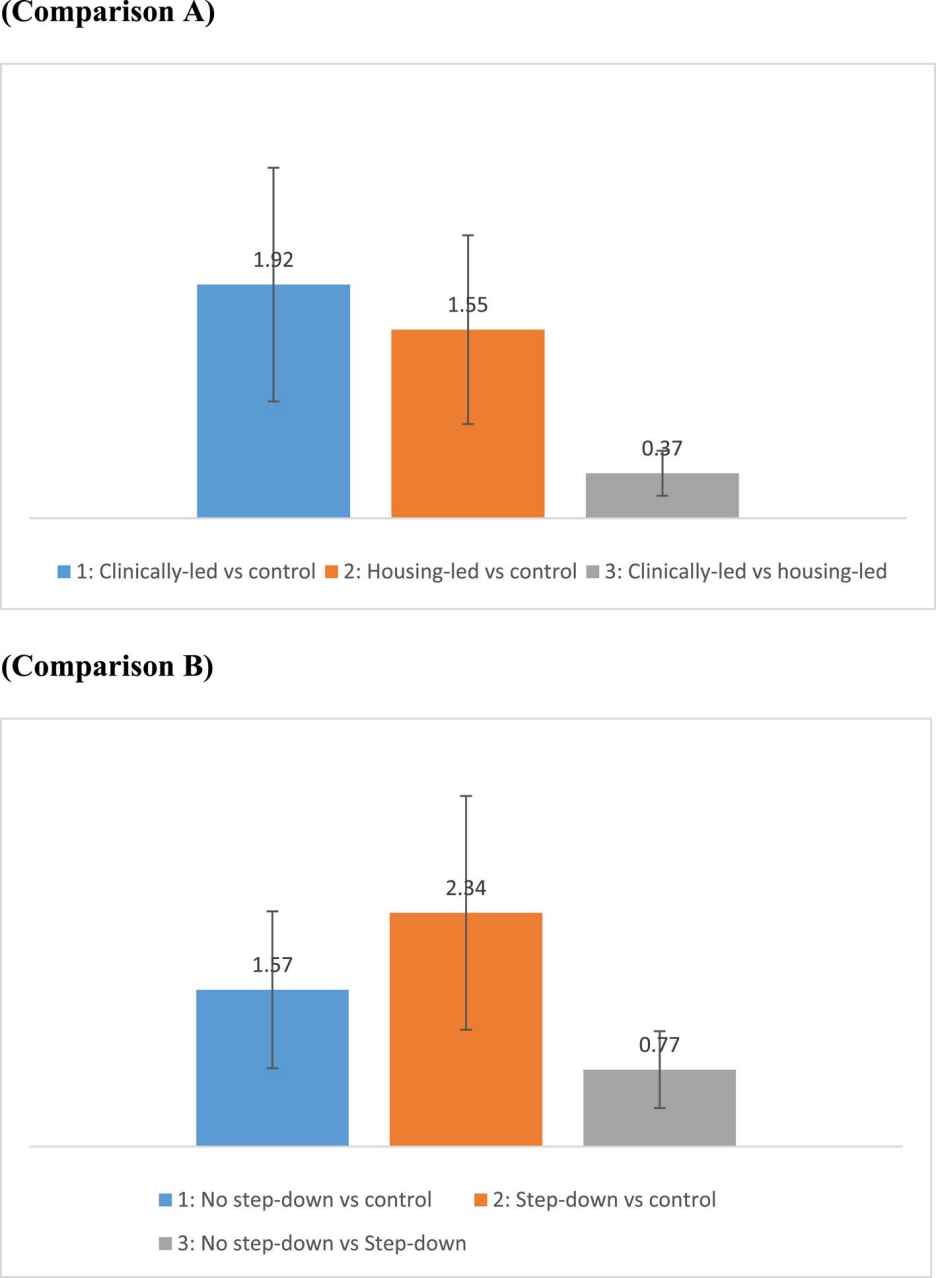
Difference in annual bed days avoided per patient. Note: Main analysis: the vertical bars report the difference in annual bed days per patient between HHD schemes and standard care. Comparison A (Difference 1: Clinically-led vs control; Difference 2: Housing-led vs control; Difference 3: Clinically-led vs housing-led); Comparison B (Difference 1: No step-down vs control; Difference 2: Step down either community-based or residential vs control; Difference 3: No step down vs step down). More information on the distribution of the variables from different groups is provided in Table 1. Sensitivity analysis: the error bar represents the variability of data when varying the annual bed days per scheme up to − 50%/+ 50% (whilst keeping the annual bed days per standard care constant)

Figure 1 shows the difference in annual bed days per patient. The differences in bed days between the schemes were similar for all comparisons between clinically-led, housing-led schemes and control (the differences were 1.92 days, 1.55 days and 0.37 days; clinically-led vs control, housing-led vs control; clinically-led vs housing-led respectively), and no step-down, with step-down sites and control (the differences were 1.57 days, 2.34 days and 0.77 days).

#### Costs per patient for each readmission

**Fig. 2:**
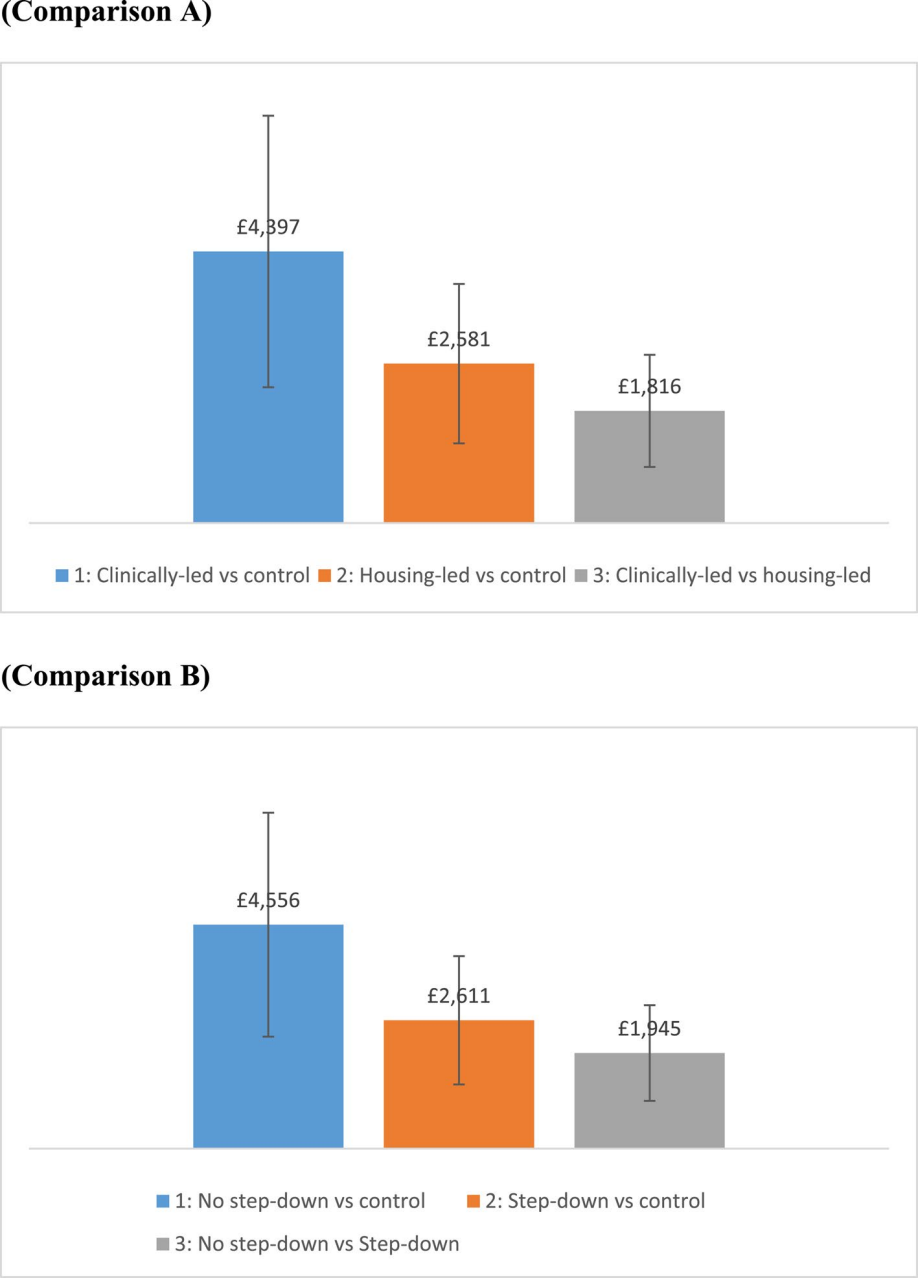
Difference in total annual NHS costs for all readmissions per patient (between HHD schemes and standard care). Note: Main analysis: the vertical bars report the difference in NHS costs per patient between HHD schemes and standard care. We reported costs for all re-admissions [i.e. re-admitted for the same problem] including the sum of elective, emergency and other readmissions. Comparison A (1: Clinically-led vs control; 2: Housing-led vs control; 3: Clinically-led vs housing-led); Comparison B (1: No step-down vs control; 2: Step down either community-based or residential vs control; 3: No step down vs step down). Sensitivity analysis: the error bar represents the variability of data when varying the annual NHS costs per patient by − 50%/+ 50% (whilst keeping the annual bed days per standard care constant)

Figure 2 shows the difference in annual NHS costs per patient for a range of readmission types (elective, emergency readmissions and other readmissions). When costs for all readmissions were combined, patients with experience of homelessness across the specialist HHD intervention groups were likely to use more resources compared with standard care, in terms of higher rates of readmission and, in turn, increased hospital costs.

Clinically-led schemes presented greater annual NHS costs per patient (vs. standard care) compared with housing-led schemes (the differences were £4,397, £ 2,581 and £1,816). Similarly, ‘no step-down schemes’ are likely to report greater annual NHS costs compared with step-down (the differences were £4,556, £2,611 and £1,945). The difference between intervention groups was statistically significant at the 0.01 level.

Clinically-led schemes are likely to report higher costs for elective readmissions compared to housing-led schemes (the differences were £2,979, £1,534 and £1,445). Similarly, schemes with no step-down services are likely to report a similar trend in data for elective readmissions compared to schemes with step-down services (the differences were £3,213, £1,339 and £1,874).

Clinically-led services have slightly higher costs associated with emergency readmissions than housing-led schemes (the differences were £1,477, £1,069 and £408). The use of step-down results in slightly lower costs associated with emergency readmissions than no step-down (the differences were £1,401, £1,294 and £107).

## Discussion

### (Specialist versus standard care)

Data from our comparative analysis of different HHD schemes for patients with experience of homelessness in England show that specialist care is likely to be more effective than standard care and to be cost-effective in comparison with standard care, as patients receiving specialist care use fewer hospital bed days per year. This suggests that HHDs may perform better than standard care at avoiding unnecessary and unpleasant hospital stays while securing better management of patient flow and preventing delayed discharges. Our analysis of NHS England figures for delayed transfers of care confirmed this [8].

Overall, specialist care may increase NHS costs by increasing access to appropriate elective care. Patients with experience of homelessness using specialist care have more readmissions to hospital compared to patients with experience of homelessness who use standard care. About the increased number of planned readmissions for elective care, our opinion is that these findings are an indication of the effectiveness of specialist care, which can improve the level of access to – and use of – elective healthcare services needed by patients with experience of homelessness [15].

### (Clinically-led versus housing-led)

When considering resources to be invested per bed day avoided housing-led (uniprofessional) teams are more cost-effective than those that are clinically-led (multidisciplinary). Our realist programme theory about the assumed benefits of clinically-led multidisciplinary team working was challenged by this. However, when looking at bed days avoided, we were unable to control for contrasting levels of patient need. A possible explanation could be that the clinically-led schemes are working with patients with more complex and/or severe needs [8]. As a result such schemes would appear less effective and cost-effective when compared with the housing-led schemes. Also, we could not control for context and different access to housing and other community services. Since the vast majority of clinically-led teams serve larger cities (because they need to look after 200 or more patients with experience of homelessness a year to be viable) it could be that they are located in areas with scarce resources, poor integrated care planning, and limited opportunity to operate within and across geographic boundaries – all of which can make it more difficult to achieve good outcomes.

Clinically-led teams report higher costs for elective readmissions as compared to housing-led ones, where there is a cost-saving against standard care. As mentioned above, we consider this to be a positive outcome (in terms of better access to planned follow-up care). We assume that more planned follow-up care is secured via clinically-led advocacy where the clinician team of GPs/nurses in the hospital may have easier access to outpatient and other clinical services than housing workers. However, we found that step-down services are also cost-saving for elective readmissions when we anticipated that continuing support after leaving the hospital would increase access to elective health care. A possible explanation could be that the majority of HHD schemes with step-down are housing-led so may not provide access to clinically-led advocacy.

If we take the above assumptions into account and consider emergency readmissions only, the difference in cost-effectiveness between clinically-led and housing-led schemes is minimal.

### (Step-down versus no step-down)

In terms of resources invested per hospital bed day avoided, HHD schemes with direct access to step-down are more cost-effective than schemes with no access to step-down. Access to step-down results in slightly lower costs associated with emergency readmissions than schemes without step-down.

Clinically-led teams do not provide the same level of access to step-down as housing-led schemes: this is because they focus on improving the quality of in-patient care and discharge coordination. Almost all the housing-led schemes work ‘in-reach’ into the hospital and this enables them to provide floating support after discharge until community services are in place. The association with intermediate care and the provision of continuity of support may explain the better performance of housing-led schemes on some measures.

Nevertheless, while we have highlighted the importance of clinically-led teams in increasing access to planned health care, it is also important to note just how effective and cost-effective relative to standard care uniprofessional housing-led HHD schemes are. Most likely this reflects the value of good quality ‘floating support’ as they can bridge the gap between hospital and community [15].

### Strengths and limitations

The main strength of our analysis is that this study is the largest evaluation of homeless hospital discharge schemes taking place in England (and, to our knowledge, internationally). The study involved 17 different schemes from different and diverse localities across England. No other study in this field has adopted the same approach using multiple data linkage to assess the impact of hospital discharge service delivery on different aspects of cost-effectiveness for the NHS. There are several limitations to this study. The most important of these was the lack of real controls and the need to use proxy data from an earlier RCT as the comparison group [10]. There is a risk that the make-up of the control group is different from some or all of the intervention groups; there may be some systematic differences in both the patient characteristics in each intervention group and the setting (e.g. urban vs. rural).It is not clear if there are differences between the characteristics of patients in the study and the control. Outcomes amongst patients enrolled in the control arm of a trial may be better than real-world outcomes of untreated groups. This may underestimate the effect of interventions. We were not able to fully control for the broader systems of care and housing for the different groups, and for any differences in quality between the different schemes and, hence, their potential impact on our analyses. Also, there may be a caveat about how closely the estimated costs reflect actual resource use. When costing hospital spells, we assumed the same tariff for spells of different duration. This is from a commissioner perspective. From a provider perspective the costs—and underlying use of resources such as nursing staff time—are likely to be high for a longer stay than a shorter stay, other factors equal. More details on the study limitations about the control group and other issues are reported elsewhere [8].

While our CCA analysis provides valuable information on the efficiency of resource allocation and helps decision-makers compare alternative interventions, access to information in multiple exclusion homelessness is extremely challenging and it does not determine whether the intervention directly causes the observed outcomes. Establishing causality requires more rigorous study designs, such as randomised controlled trials or adjustments to address possible underlying biases due to data coming from non-randomised study designs which were not possible in this environment [16]. The lack of patient level information for the control prevented application of statistical tests to assess the statistical significance of cost and outcome differences. Also, our data has a limitation in that service delivery costs and QALY data were unavailable for all 17 HHD scheme sites. Consequently, our primary analyses could not include a cost-effectiveness model to compare the success of the 17 HHD schemes against the National Institute for Health and Care Excellence (NICE)’s cost per QALY thresholds [17]. However, in more detailed case analyses, we gathered valuable information on service delivery and QALYs, which was incorporated into additional economic models which considered both NHS (supplementary material, Appendix 1) and broader public budget perspectives [18]. This detailed analysis is limited to a subset of only three sites and cannot be extrapolated to the larger group of 17 schemes. Future research should involve the standardised measurement of service delivery costs and health outcomes and their variation across sites and time. The study reports on the situation in England. The findings may not be directly relevant to healthcare systems in other countries.

## Conclusions

This evaluation provides good evidence for commissioners that specialist care for the discharge from hospitals of people experiencing homelessness – especially specialist care encompassing step-down intermediate care—is more effective than standard care and is cost-effective in comparison with standard care. Our realist hypothesis was refined to highlight the value of embedding good quality'housing-led'support as an integral component of multi-disciplinary work to secure the safe, timely discharge of patients with experience of homelessness. Our findings support the creation of a toolkit for commissioners and practitioners on developing specialist integrated homeless health and care services [19].

## Supplementary Information

Supplementary Material 1.

## Data Availability

Data that support the findings of this study are available in the text and supplementary material of this article.

## Abbreviations

COVID- 19: Coronavirus disease 2019
CCA: Cost-consequence analysis
DH: Department of Health
DHSC: Department of Health and Social Care
EDs: Emergency Departments
GPs: General Practitioners
HHD: Homeless hospital discharge
HHDF: Homeless Hospital Discharge Fund
HES: Hospital Episode Statistics
NICE: National Institute for Health and Care Excellence
QALYs: Quality-adjusted life years
