# Supplementary material for: The cost-effectiveness of specialist hospital discharge and intermediate care services for patients who are homeless
Source: BMC Health Serv Res. 2025 Jun 3;25:794. doi: 10.1186/s12913-025-12704-x (PMC12135440; doi:10.1186/s12913-025-12704-x)
Supplement: Supplementary file 1 — Supplementary Material 1. [file 12913_2025_12704_MOESM1_ESM.docx]

## Supplementary material

**Appendix 1: Secondary analysis: Comparisons of costs and outcomes of three schemes with different configurations**

1. ***Aims***

We wanted to consider the costs effectiveness of specialist hospital discharge and intermediate care (support after discharge) services for people who are homeless in England, considering impacts on health outcomes (quality adjusted life years) looking at three different ‘in patient care coordination and discharge planning’ configurations. We also wanted to estimate the costs of these three service models and make them available to local policy decision-makers who might be considering providing similar services.

1. ***Methods***

The three schemes included in the secondary analysis presented different service configurations as it follows:

(Configuration 1) This involves a clinically led scheme providing patient in-reach and discharge coordination, without a 'step-down' service. Typically led by nurses or general practitioners (GPs), these schemes include in-reach activities such as hospital ward rounds.

(Configuration 2) This configuration encompasses clinical and housing in-reach, discharge coordination, and access to 'step-down' intermediate care.

(Configuration 3) This involves a housing-led scheme primarily focused on offering accommodation to individuals with a history of homelessness upon discharge from the hospital. This configuration includes a team of housing support workers providing patient inreach, discharge coordination, and community-based step-down services.

A cost consequence model was applied.

The outcomes included quality adjusted life years (QALYs) and were calculated from EQ-5D data collected at baseline and 12-month follow-up.^1^ The calibration of the QALY scores came from Hewett et al. (2016).^2^ We considered the annual economic consequences on NHS looking at hospital readmissions, as reported in the main analysis. Also, we collected additional information on annual service delivery costs to cover staff salaries, their training and subsistence expenses, overheads and other items.^3^ QALY outcomes, hospitalisation costs and service delivery costs were compared between configurations 2/3 and configuration 1 (standard care).

QALY (at baseline, index discharge, and 12-month follow-up) data for the secondary analysis were collected between 1 January 2015 and 30 September 2017. Annual intervention cost data^4^ refer to 2017 figures. Cost A summary of the economic model for the secondary analysis is presented in Table S1.

**Table S1: Summary of the cost consequences model for the secondary analysis**

| **Parameter** | **Secondary analysis, looking at three schemes – is presented to fill in the main gap in data access for the 17 schemes** |
| --- | --- |
| Question to be answered | What are the costs consequences of specialist hospital discharge and intermediate care (support after discharge) services for people who are homeless in England? |
| Alternatives compared | The analysis includes a comparative focus on three configuration types:  1: clinically-led scheme without step-down (as per standard care, see main analysis)  2: clinically-led scheme with step-down  3: housing-led scheme with step-down.  QALY outcomes and service delivery costs were compared between configurations 2/3 and configuration 1 (standard care). Aggregate level data on the use of NHS resources for ‘standard care’ were sourced from Hewett et al. (2016).^2^ |
| Type of economic evaluation | Cost-consequence analysis |
| Country setting | England |
| Perspective on costs | NHS |
| Cost data and sources of evidence | We consider the economic consequences on NHS [hospital readmissions, see main analysis] as well as the annual service delivery costs missing from the main analysis of the 17 schemes. Estimates were extracted from local audit; for details see Table S2). |
| The base year for calculating costs/prices | All costs are in 2017 prices (the period over which participants were studied and the resources were used). |
| Currency unit | British pound (£). |
| Effectiveness outcomes | Here we present the QALY outcome missing from the analysis of the 17 schemes. |
| Effectiveness data and sources of evidence | Intervention groups: Estimates were collected using EQ-5D questionnaires^3^ and  Standard care: Estimates were extracted from Hewett et al. (2016)^2^. |
| Time horizon and discounting | 12 months. The cost and consequences were considered for a limited period of 12 months, so no discounting needed. |
| Statistical analysis | The cost and effectiveness of the intervention groups was established conducting a comparative analysis by using summary statistics for the control group and individual-level data for the intervention groups. We did not get access to the original cost and outcome dataset from the trial data (with patient-level information) and we could not test for group differences. |
| Sensitivity analysis | We varied individual costs and outcomes by a given amount (up to +/- 50%) and examined the impact on model results. |

1. ***Results: The effect of HHD schemes on QALYs***

Both configurations 2 (scheme with step-down) and 3 (housing-led scheme) present better QALY outcomes compared with configuration 1 (clinically-led scheme without step-down as per standard care) (Figure 3). Configuration 3 presents the highest number of QALY gains (compared with standard care; Difference 1: Configuration 2 - Configuration 1= 0.29* vs. Difference 2: Configuration 3 - Configuration 1= 0.17* vs. Difference 3: Configuration 3- Configuration 2= 0.12*; *p<0.05).

**Figure S1: Difference in annual QALYs per patient between configurations (secondary analysis)**

Note: The vertical bars report the difference in annual QALY gains per patient between configurations. Configuration 1: clinically-led scheme without step-down as per standard care; Configuration 2: Scheme with step-down; Configuration 3: Housing-led scheme. Difference 1: Configuration 2 -Configuration 1; Difference 2: Configuration 3-Configuration 1; Difference 3: Configuration 3 - Configuration 2. **Sensitivity analysis**: the error bar represents the variability of data when varying the annual QALYs by -50%/+50% (whilst keeping the annual QALYs per configuration 1 constant).

**Figure S2: Difference in annual NHS service delivery costs per patient between configurations (secondary analysis)**

Note: The vertical bars report the difference in annual NHS service delivery costs per patient between configurations. Configuration 1: clinically-led scheme without step-down as per standard care; Configuration 2: Scheme with step-down; Configuration 3: Housing-led scheme. Difference 1: Configuration 2 -Configuration 1; Difference 2: Configuration 3-Configuration 1; Difference 3: Configuration 3 - Configuration 2. Service delivery costs for the individual configurations are reported in Table S2. **Sensitivity analysis**: the error bar represents the variability of data when varying the annual service delivery costs for configurations 2/3 by -50%/+50% (whilst keeping the annual service delivery costs per configuration 1 constant).

**Sensitivity analysis.** The error bars in Figures 1 to 4 represent the variability of data when varying the difference in costs and outcomes (compared with standard care) by -50%/+50%. For example, figure 1 shows that for schemes with no step-down, if we decrease the difference in annual bed days per patient by 50% (-1.57-1.57*0.5=-2.35), it will be similar to the difference in annual bed days reported for schemes with step-down either community-based or residential (-2.34).

1. ***Results: The difference in NHS service delivery costs between HHD schemes***

More NHS money would need to be invested to deliver both configurations 2 (scheme with step-down) and 3 (housing-led scheme) compared with configuration 1 (figure 4). Configuration 2 would be the most expensive (annual service delivery costs per patient £5400) followed by configuration 3 (£2000) and configuration 1 (£800; see supplementary material, appendix 2).

## Table S2 Annual NHS service delivery costs (year 2017)

| Items | Configuration 1, Control  ('*Standard care’*) | Configuration 2 | Configuration 3 |
| --- | --- | --- | --- |
|  | **(Provision for 206 users from the RCT)** | **(Provision for 80 users)** | **(Provision for 88 users)** |
| 1. Paid staff – total | £123,500 | £264,000 | £122,500 |
| 2. Staff training, travel and subsistence expenses (e.g. for outreach activities) | £12,300 | £3,500 | £10,000 |
| 3. Overhead costs – non-staff | £22,400 | £105,200 | £7,700 |
| 4. Overhead costs – staff | £10,300 | £1,500 | £3,200 |
| 5. Capital overheads | £2,600 | £58,900 | £29,300 |
| Total yearly costs, 2017 | £171,000 | £433,200 | £172,600 |
| Average cost per patient experiencing homelessness* | £800 | £5,400 | £2,000 |

Note: **Configuration 1** (**clinically-led scheme)**: provided patient in-reach and discharge coordination, with no 'step-down' service. These schemes are usually nurse or general practitioner (GP)-led and include in-reach (hospital ward rounds) and discharge coordination. **Configuration 2** (**Step-down scheme)**: it comprised clinical and housing in-reach, discharge coordination and access to ‘step-down’ intermediate care. **Configuration 3** (**Housing-led scheme)**: which primarily focused on providing accommodation to individuals with experience of homelessness on discharge from hospital. They included a group of housing support workers providing patient in-reach, discharge coordination and community-based step-down. Please note that public sector perspective: intervention costs incurred by the broader public sector (including NHS, social care and housing) are presented elsewhere.^14^ * We relied on expert opinion to calculate an average estimate per patient experiencing homelessness assuming that equal resources were allocated to each patient they provided care for in the year.

1. ***Conclusion***

The secondary analysis is a fuller cost-effectiveness analysis than the main analysis, where not all costs could be included, and the outcomes are not ‘final’ outcomes. With this analysis we confirmed that these specific schemes are likely to be cost-effective compared with standard care. Also, we found better QALY outcomes, but results are not generalisable to all 17 schemes.

1. ***References***
2. NHS Digital. Hospital Episode Statistics (HES). 2019. https://digital.nhs.uk/data-and-information/data-tools-and-services/data-services/hospital-episode-statistics#:~:text=Hospital%20Episode%20Statistics%20(HES),at%20NHS%20hospitals%20in%20Englan . Accessed 21 May 2024.
3. Hewett N, Buchman P, Musariri J, Sargeant C, Johnson P, Abeysekera K, et al. Randomised controlled trial of GP-led in-hospital management of homeless people ('Pathway'). Clin Med (Lond). 2016;16:223-9.
4. Cornes M, Aldridge RW, Biswell E, Byng R, Clark M, Foster G, et al. Improving care transfers for homeless patients after hospital discharge: a realist evaluation. Health Services Delivery Research 2021;9:17.
5. Tinelli M, Wittenberg R, Cornes M, Aldridge RW, Clark M, Byng R, et al. The economic case for hospital discharge services for people experiencing homelessness in England: An in-depth analysis with different service configurations providing specialist care. Health Soc Care Community. 2022;30:e6194-e6205.

### Appendix 2: Resource items and their unit costs

| **Type of cost** | **Configurations 1 and 2** | **Configuration 3** | **Unit cost (2017)** | **Source of unit costs** |
| --- | --- | --- | --- | --- |
| **Healthcare costs** | Hospital Admissions (average) | Hospital Admissions (average) | £1,783 | PSSRU 2017 unit costs of health and social care |
|  | Elective inpatient stays | Elective inpatient stays | £3903 | PSSRU 2017 unit costs of health and social care |
|  | Emergency admission* | Emergency admission* | £1,074 | PSSRU 2017 unit costs of health and social care |
|  | Visits to A&E | Visits to A&E | £157 | PSSRU 2017 unit costs of health and social care |
|  | Hospital outpatient attendances | Hospital outpatient attendances | £137 | PSSRU 2017 unit costs of health and social care |
|  | GP visits (GP contact per patient contact lasting 9.22 minutes) | GP visits (GP contact per patient contact lasting 9.22 minutes) | £38 | PSSRU 2017 unit costs of health and social care |
|  |  | 999 Ambulance (average all callouts) | 205 | PSSRU 2017 unit costs of health and social care |
| **Mental health care costs** | Admission to a mental health hospital (Mental health care clusters per day) | n/a | £404 | PSSRU 2017 unit costs of health and social care |
|  | contact with Mental health community provision (Mental health specialist teams (per care contact)) | n/a | £172 | PSSRU 2017 unit costs of health and social care |
|  | Stay in a local authority care home for people with mental health problems [average duration 4*12 weeks] | n/a | £786 | PSSRU 2017 unit costs of health and social care |
|  | Access to local authority social services day-care for people with mental health problems | n/a | £35 per client attendance | PSSRU 2017 unit costs of health and social care |
|  | n/a | Mental health services for the 1 year (average based on Derby data) | £2,670 | PSSRU 2017 unit costs of health and social care |
| **Drug and alcohol treatment costs** | Substitute prescriptions (methadone) | n/a | £55 a week | Gossop 2015 |
|  | detox and rehab centre stay | n/a | Average £417 | PSSRU 2017 unit costs of health and social care |
|  | one-to-one contact with a drug/alcohol treatment team | n/a | Average £125 | PSSRU 2017 unit costs of health and social care |
|  | Group session contacts with a drug/alcohol treatment team | n/a | Average £16 | PSSRU 2017 unit costs of health and social care |
|  | n/a | Drug and alcohol treatment services for the 1 year (average based on Derby data) | £1,061 | PSSRU 2017 unit costs of health and social care |
| **Housing costs** | Rough sleeping | Rough sleeping | £7,900 | Ashton and Hempenstall (2009) |
|  | Direct access hostel (night) | n/a | £48 | Ashton and Hempenstall (2009) |
|  | The second stage supported accommodation (Semi-independent accommodation) | n/a | £27 per night | MEAM 2014 |
|  | Own social tenancy | n/a | £69 per week | Average from MEAM 2014 |
|  | Own private rented sector tenancy | n/a | £100 per week | Average from MEAM 2014 |
|  | Room in shared private rented sector property | n/a | £65 per week | Average from MEAM 2014 |
|  |  | Homelessness investigation and decision | £6639 | Shelter’s “Value for Money in Housing Options and Homelessness” Report (2010) |
| **Criminal justice costs** | Arrest by police | n/a | £2,130 | Think Family 2010 |
|  | Other police contact | n/a | £17 | Winsor (2011) https://www.gov.uk/police-pay-winsor-review |
|  | Magistrate court attendance | n/a | £995 | Average from MEAM 2014 |
|  | Crown court attendance | n/a | £11,245 | Average from MEAM 2014 |
|  | Nights in prison/Nights in police custody | n/a | £75 | Average from MEAM 2014 |
| **Social care costs** | Comprehensive Clinical Assessment | n/a | £55 | PSSRU 2017 unit costs of health and social care |
|  | n/a | OT assessment | £35 | PSSRU 2017 unit costs of health and social care |
|  | Visit with a social worker | Visit with a social worker | £55 | PSSRU 2017 unit costs of health and social care |
|  | Night in residential care | Night in residential care | £543 per week | PSSRU 2017 unit costs of health and social care |
| **Social benefits** | Employment Support Allowance | n/a | £73.10 per week | Benefits entitlement |
|  |  |  |  | Gov.uk |
|  | Personal Independence Payment (PIP) | n/a | £57.30 per week | [Benefits entitlement](https://www.gov.uk/browse/benefits/entitlement) |
|  | (Daily living - standard rate) |  |  | Gov.uk |
|  | Disability Living Allowance (DLA) for adults | n/a | £57.30 per week | [Benefits entitlement](https://www.gov.uk/browse/benefits/entitlement) |
|  | [Frequent help or constant supervision during the day, supervision at night or someone to help you while on dialysis] |  |  | Gov.uk |
|  | Universal credit | n/a | £317.82 per month | [Benefits entitlement](https://www.gov.uk/browse/benefits/entitlement) |
|  |  |  |  | Gov.uk |
| **State pension** |  | n/a | £125.95 per week for those reaching SPA before April 2016 (SPA = state pension age) | [Benefits entitlement](https://www.gov.uk/browse/benefits/entitlement) |
|  |  |  |  | Gov.uk |
| **Service delivery costs** | Conf.2 Site delivery costs were calculated using costing information collected from the site | Service delivery costs were calculated using costing information collected from the site | See Table 2, main text | Hewett et al. (2016)  Local site data  Local site data |
